# Clinical Importance of Magnification in the Assessment of Colorectal Lesions

**DOI:** 10.1093/jcag/gwaa036

**Published:** 2020-11-18

**Authors:** Robert Bechara, Paul Manley

**Affiliations:** 1 Department of Medicine, Division of Gastroenterology, Queen’s University, Kingston Health Sciences Center, Kingston, Ontario, Canada; 2 Department of Pathology, Queen’s University, Kingston Health Sciences Center, Kingston, Ontario, Canada

A 56-year-old man had a 1.5 cm Paris 1s+2c rectal lesion detected on fecal immunochemical test-positive colonoscopy. Examining the lesion using optical enhancement mode 1 (OE-1), which utilizes wavelengths 415 nm and 540 nm, highlights the microvasculature. This revealed a NICE III lesion, indicating cancer (Figure 1) (1).

**Figure 1.**
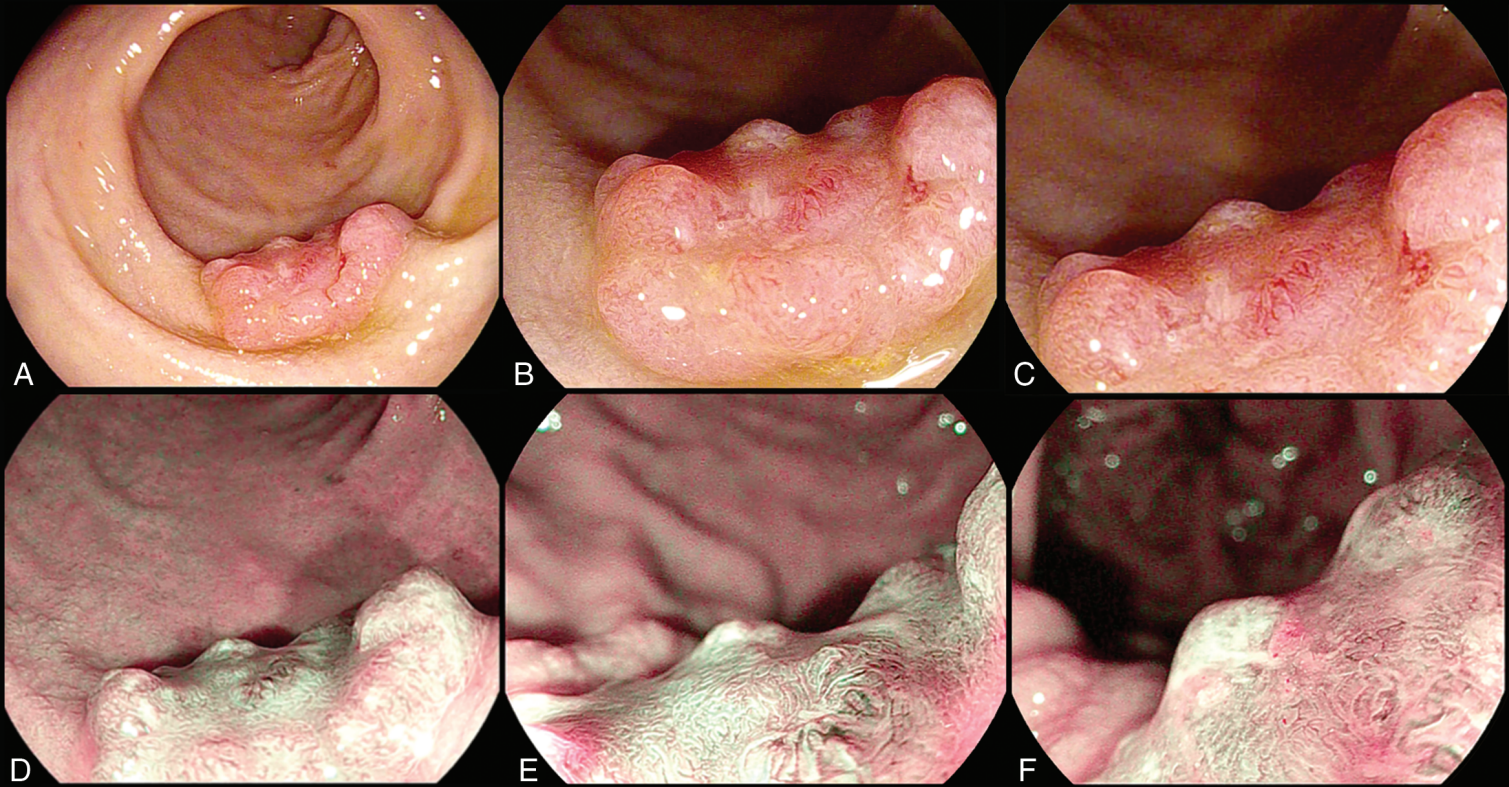
Rectal Lesion. White light images of lesion: (**A**) distant view, (**B**) near view, (**C**) low magnification. OE-1 images of lesion: (**D**) distant view, (**E**) low magnification, (**F**) medium magnification.

The NICE classification is advantageous in its simplicity and the fact that magnification is unnecessary. However, a drawback is that neoplastic lesions are only classified into two categories: NICE II (adenoma) and NICE III (invasive cancer). To address this limitation, the Japan NBI Expert Team (JNET) created the JNET classification where NICE II lesions are subclassified using magnification: JNET 2A (low-grade adenoma) and JNET 2B (high-grade dysplasia or superficially invasive cancer) (2).

Under magnification, the lesion of interest was classified as JNET 2B, which suggests superficial neoplasia that is potentially resectable endoscopically. Considering the central depression along with microvasculature findings, the lesion was staged as a rectal cancer. MRI staged the lesion as a T1-T2, and CT was negative for any metastatic disease.

An endoscopic submucosal dissection (ESD) was completed and a curative R0 resection was achieved with final pathology of a well-differentiated adenocarcinoma with superficial submucosal invasion (<_ 1000 µm) without tumor budding or lymphovascular invasion (Figure 2).

**Figure 2.**
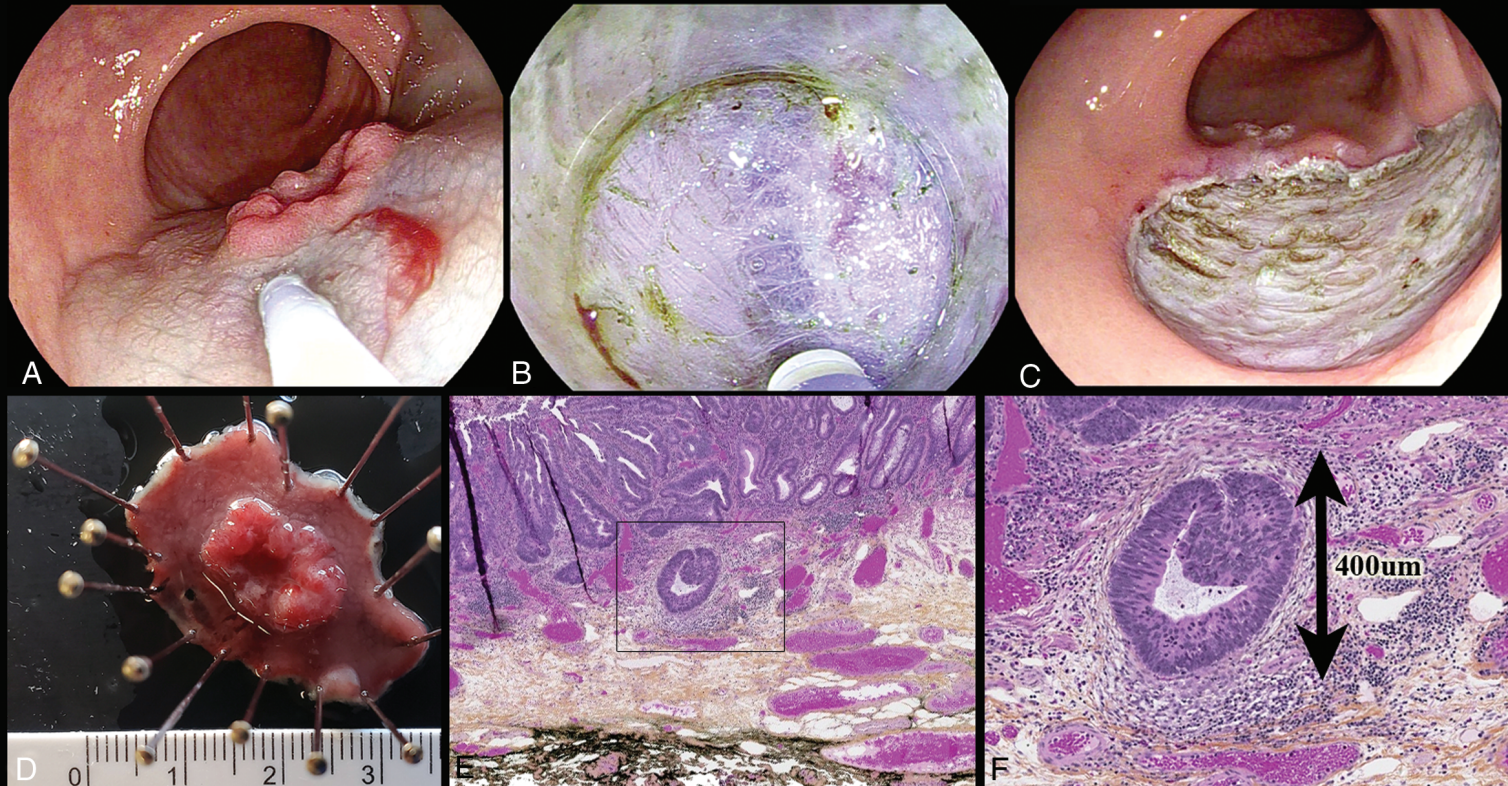
Resection and pathology: (**A**) submucosal injection of lesion; (**B**) ESD: muscularis propria on the left and submucosa/mucosa to the right; (**C**) defect post-ESD; (**D**) pined gross specimen; (**E**) 20× with hematoxylin phloxine saffron (HPS) stain: tubular adenoma with high-grade dysplasia and focus of superficial adenocarcinoma; (**F**) 40× HPS stain: focus of submucosal cancer with penetration depth of 400 um.

## References

[1] Hewett DG, Kaltenbach T, Sano Y, et al. Validation of a simple classification system for endoscopic diagnosis of small colorectal polyps using narrow-band imaging. Gastroenterology 2012;143(3):599–607.e591. 22609383 10.1053/j.gastro.2012.05.006

[2] Sumimoto K, Tanaka S, Shigita K, et al Clinical impact and characteristics of the narrow-band imaging magnifying endoscopic classification of colorectal tumors proposed by the Japan NBI Expert Team. Gastrointest Endosc 2017;85(4):816–21. 27460392 10.1016/j.gie.2016.07.035

